# General practitioners' self-perceived ability to recognize severity of common mental disorders: an underestimated factor in case identification?

**DOI:** 10.1186/1745-0179-2-21

**Published:** 2006-08-30

**Authors:** Ingrid Olssøn, Arnstein Mykletun, Alv A Dahl

**Affiliations:** 1 Department of Psychiatry, Innlandet Hospital Trust, 2300 Hamar, Norway Faculty of Medicine, University of Oslo, 0316 Oslo, Norway; 2 Research Centre for Health Promotion, University of Bergen, 5015 Bergen, Norway; 3 Norwegian Institute of Public Health, Division of Epidemiology, Department of Mental Health, Oslo, Norway; 4 Department of Clinical Cancer Research, Rikshospitalet-Radiumhospitalet Trust, 0310 Oslo, Norway

## Abstract

**Background:**

Several studies have shown that general practitioners (GPs) under-diagnose common mental disorders, and that training courses hardly improve this practice. The influence of GPs' self-perceived ability to recognize the severity of such disorders on these facts has not been investigated. This study explores: 1) GPs' perceived ability to recognize major depressive episode (MDE) and generalized anxiety disorder (GAD) in their patients; 2) The GPs' observed ability to recognize severity of these disorders; and 3) If the observed ability to recognize severity is associated with their perceived ability.

**Methods:**

In a cross-sectional design 40 Norwegian GPs examined 15 – 28 patients each (total N = 724). The GPs' rated their perceived ability to recognize MDE and GAD on a four-point Likert-scale. The GPs' observed ability to recognize severity was defined as the mean of the correlations between the GPs rating of Clinical Global Impression-Severity Scale and the diagnostic reference standards for MDE and GAD filled in by patients.

**Results:**

Twenty-two GPs considered their perceived ability to recognize MDE as rather good, and the other 18 as moderate/bad. For GAD 12 GPs' perceived their ability as rather good, while 28 considered their ability to be moderate/bad. The observed ability to recognize severity concerning MDE was 0.63 and concerning GAD 0.45. There was no significant association between GPs' perceived and observed abilities to recognize MDE (p = 0.19) and GAD (p = 0.34)

**Conclusion:**

This study found a discrepancy between the GPs' perceived and observed ability to recognize common mental disorders. The lack of association between GPs' perceived and observed ability to recognize such disorders indicate low understanding of own recognition abilities. This might contribute to explain the low effectiveness of interventions aimed to increase GPs' abilities to recognize mental disorders.

## Background

Despite most persons with anxiety disorder and/or depression do not attend their GP [1,2] major depressive episode (MDE) and generalised anxiety disorder (GAD) are common mental disorders in general practice. Studies have shown that general practitioners (GPs) under-identify these disorders when compared to standard categorical diagnoses made by structured interviews or by psychiatrists [3,4]. Educational programs introduced to improve GPs' recognition and management of these mental disorders has not been very successful and only short-lived effects have been reported [5-10]. A review [5] pointed out that most studies emphasize what GPs 'objectively' need to know, and do not take into account any perceived need or motivation for GPs to improve their recognition skills. Time pressure and heavy caseloads and also GPs' 'expert role' in the consultation room could contribute to a low awareness of their own ability to recognize common mental disorders.

The studies do not clarify possible reasons for the low effectiveness of interventions aimed to improve GPs' abilities to recognize common mental disorders. One explanation might be that the GPs' perceive their ability to identify cases as 'good enough'. Dowrick et al [11] tested the hypothesis that questionnaire measures of the GPs' confidence in identifying depression predicted their ability to identify depression in patients. The measure of GPs' perceived ability to recognize was the "Identification of depression" component of the Depression Attitude Questionnaire [12], and no significant associations with the patients' scores on the General Health Questionnaire (observed ability) were found. To the best of our knowledge there are no published studies exploring the association between perceived and observed ability concerning anxiety disorders.

On this background we wanted to investigate the following research questions in a sample of Norwegian GPs and their patients: 1) What is the GPs' self-perceived ability to recognize MDE and GAD in their patients? 2) What is the GPs' observed ability to recognize severity of these disorders? and 3) To describe the association between GPs' observed ability to recognize severity and their self-perceived ability. We held the hypothesis that GPs' observed ability to recognize severity would be positively associated with their perceived ability.

## Methods

### Design and material

This study is based on the Norwegian part of a cross-sectional study of GPs in relation to MDE and GAD carried out in Germany, Scandinavia and Finland [13,14]. Essential features of the design were: 1) Pre-study sampling of the GPs' demographic data, their perceived recognition ability, and routine management of GAD and MDE. 2) During three consecutive days in September 2001 all the GPs' patients were invited to take part in a study and to fill in a questionnaire concerning demographic characteristics, reason for and number of visits to GPs last year, as well as the Generalized Anxiety Questionnaire (GAS-Q) and the Depression Screening Questionnaire (DSQ). 3) Blind to the patients' self-ratings, the GPs rated the clinical severity of eventual GAD and MDE in their patients on the Clinical Global Impression-Severity scale (CGI-S). Detailed information concerning design and methods have been described earlier [13].

Patients excluded from the study were those below 16 years of age, who had language difficulties, were considered to require help to complete the questionnaires, or who only came for a prescription or for an emergency.

The national study leader coordinated the study that was sponsored by the pharmaceutical company Wyeth Norway Ltd. This implied that the representatives of Wyeth Ltd recruited a convenience sample of GPs all over Norway among the GPs registered in the representatives' databases of their districts, which in practice included close to all GPs in Norway. The procedural instructions to the GPs were given in writing, and no special training of the GPs for the study was arranged. The company brought the material for the study to the GPs and collected the questionnaires, but otherwise took no active part in the study.

### The patient rated questionnaires

The patients gave information concerning demographic data, their sick leave status and number of visits to a GP last year. In addition they responded to the statement: "The reason for my visit to the doctor to day is?" with the opportunity to tick off among eight alternatives: three mental (depression, anxiety, sleeping problems), three somatic (physically ill, pain, accident), control/receipt renewal, and other reasons.

#### Diagnostic reference standards

The Generalized Anxiety Questionnaire (GAS-Q) was developed to diagnose GAD according to the DSM-IV [15]. The GAS-Q consists of 20 items that are filled in only if the entry question is scored positively: "During the past 4 weeks, have you been bothered by feeling worried, tense or anxious most of the time?" (Criterion A of GAD in DSM-IV). Test-retest reliability of the GAS-Q over a two-day retest period showed a kappa value of 0.74 for the GAD diagnosis. Congruent validity comparing GAS-Q diagnosis with the DSM IV algorithm for GAD of the Composite International Diagnostic Interview (CIDI) showed a kappa of 0.72 [16]. In our study the GAS-Q showed an internal consistency of Cronbach's coefficient alpha 0.91.

The Depression Screening Questionnaire (DSQ) is based on self rating [17] and was developed for the diagnosis of MDE (DSM-IV). The DSQ consists of 11 criteria-based items that are rated on a three points scale. Consistent with the DSM-IV definition, a diagnosis of MDE was assigned when at least five of the DSQ items were rated as positive. Tests of the DSQ diagnosis of MDE versus diagnosis based on a structured diagnostic interview showed a kappa 0.89 [18]. In the German part of the European study, the internal consistency of the DSQ showed a Cronbach's coefficient alpha of 0.83 [19]. In our study the Cronbach's coefficient alpha of the DSQ was 0.87.

Psychiatric classification systems like DSM-IV and ICD-10 are based on the presence/absence of diagnostic criteria. When structured interviews are used, the patients are asked for the presence or absence of such criteria by an interviewer, while when the GAS-Q and the DSQ are used, the patients themselves respond to the same criteria posed as items in a questionnaire. In our study, the diagnostic references were defined by the patients' responses to the GAS-Q and the DSQ.

### The GP rated questionnaires

The GPs gave information on their demographics, self-perceived recognition ability, and routine management of GAD and MDE, and they were allowed to use all accumulated information about their patients when doing their ratings.

#### GP's opinions on patients' severity of disorder

The Clinical Global Impression-Severity Scale (CGI-S). The GPs identified and rated cases of GAD and/or MDE on the CGI-S, which is a standardized assessment tool widely used as an outcome measure in psychiatric research [20]. The CGI-S had the following wording: "In your clinical judgement how severely does this patient suffer from MDE/GAD?" The ratings of the CGI-S are: 1 = not ill at all, 2 = a borderline case, 3 = only mildly ill, 4 = moderately ill, 5 = seriously ill and 6 = extremely seriously ill.

#### GPs' opinions on self- perceived recognition abilities

Each GP rated their self-perceived ability to recognize MDE and GAD by answering the question: "What are your self-assessed competences concerning recognition of MDE and GAD?" The GPs' responses were given separately for MDE and GAD on a four-point Likert scale (1 = bad, 2 = rather bad, 3 = moderate, 4 = rather good).

### The observed ability to recognize severity

The observed ability to recognize severity for a GP was operationalized as the mean correlation coefficient between the CGI-S score and the diagnostic reference standard (as measured with GAS-Q and DSQ) on each of his patients. The CGI-S represents GPs' opinion of the severity of the patients' MDE or GAD. The GAS-Q and the DSQ measure the patients' experience of the severity of their disorder. The observed ability to recognize is the mean of all correlation coefficients and was calculated for each GP separately. A correlation coefficient around zero indicates low recognition ability, whereas a correlation coefficient near to 1 is optimal. The observed recognition ability was included as a continuous measure in the analyses of the association to perceived ability, and was computed separately for GAD and MDE.

### Statistical analyses

The statistical analyses were carried out on the SPSS for Windows, version 13.0.

From considerations of reliability of assessments of GP's diagnostic abilities, only GP with at least 15 patients assessed for mental disorders (including valid measures of both the GP's CGI-S and the diagnostic reference standards) were included. Due to the distribution of the responses in GPs' self-perceived recognition abilities the variable was dichotomized (1 = rather good, 0 = moderate/rather bad/bad) when used as the dependent variable in the logistic regression analyses. To account for eventual confounding, GPs' demographic and professional characteristics were used as independent variables in these analyses. The observed ability to recognize for each GP was operationalized as the mean of the stratified Pearson's correlation coefficient between each GPs' CGI-S ratings and the patient-rated DSQ and GAS-Q.

Due to the hypothesis of a positive direction of the association between observed and perceived ability to recognize we have used one-tailed testing and p values < .10 were reported as significant.

### Ethics

The Committee for Medical Ethics of Health Region East of Norway approved this study. The participants delivered written informed consent after getting written information about the study. In addition to their ordinary salary, the GPs got a fee of EUR 15 per patient paid by Wyeth Norway Ltd. The company put the Norwegian dataset to our disposal without any restrictions or demands, and they did not review any drafts or manuscripts.

## Results

### Participants

In total 141 GPs rated the severity of eventual GAD or MDE with CGI-S in 1.781 patients. Among the 40 GPs who rated = 15 patients, 30 were men and 10 women, with a mean age of 45 (SD 6.6), and who had been working in primary care for a mean of 14 (SD7.5) years. On an average day the GPs consulted with a mean of 23.8 (SD 4.5) patients. Mean number of patients included in the study by each GP was 18 (SD 3.6, range 15 to 28). Findings on the GPs' demographic information and their routine management of GAD and MDE are given in Table 1. An attrition analysis showed that except for the higher number of patients included in the study, the 40 GPs did not differ significantly from the 101 not included as to gender or years in primary care service, which were the only data at disposal for such analyses.

**Table 1:** GPs' characteristics (N = 40)

Demographics		
Gender (male/female)	30/10	
Age (SD)	45.4 (6.6)	
Years in primary care office (SD)	14.0 (7.5)	
Number of patients pr week (SD)	103.0 (26.5)	

**Routine management**		

In my opinion MDE and GAD are almost the same :		
not agree	12	
partial or fully agree	28	

	***GAD***	***MDE***

Use of questionnaires or psycho- educational material :		
seldom	16	26
often	24	14
I enjoy treating patients with MDE/GAD:		
not agree	8	7
partial or fully agree	32	32

Among the GPs' patients 724 filled in the DSQ, and of them 208 rated positively on the entry question of GAS-Q, and therefore completed that form too. Among the patients 37% (n = 266) were men and 63% (n = 458) women, with a mean age of 49 (SD 18.3) and 46 (SD17.6) years, respectively. Based on the DSQ and the GAS-Q, the prevalence of MDE and GAD were 10.1% and 7.5%, respectively. Further characteristics of the patients are given in Table 2.

**Table 2:** Patients' characteristics (N = 724)

Variables	
Age, mean (SD)	47 (17.9)
Number of visits at GPs' last year (SD)	5.7 (18.2)

	***N (%) ***

Gender	
Female	458 (63)
Male	266 (37)
Civil status	
Married/paired relationship	485 (67)
Living alone	239 (33)
On sick leave:	
Yes	138 (19)
No	586 (81)
Reason for visit*	
Mental; yes	109 (15)
No	615 (85)
Somatic; yes	363 (50)
no	361 (50)
Prescription/other; yes	276 (38)
No	448 (62)

* Several alternatives possible

### Perceived ability to recognize MDE and GAD

**Figure 1:**
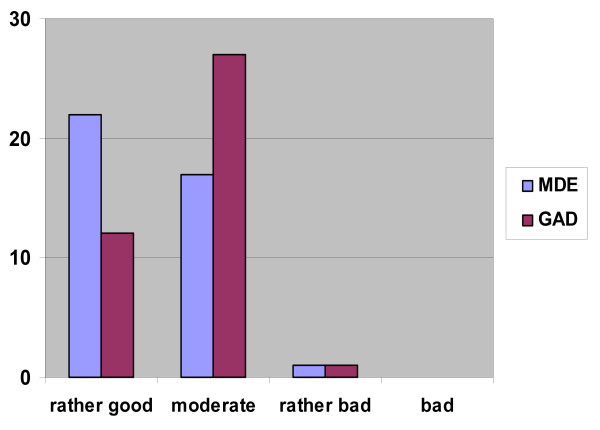
GPs' self-perceived ability to recognize common mental disorders (N = 40).

Twenty-two GPs perceived their ability to recognize MDE 'rather good' and 17 GPs perceived themselves as 'moderate' (Figure 1). In relation to GAD, 12 GPs perceived their ability to recognize the disorder as 'rather good' while 27 GPs considered themselves as 'moderate'. One GP perceived himself as having a 'rather bad' recognition ability concerning both MDE and GAD, and no GPs perceived themselves self as 'bad'. Logistic regression analyses (Table 3) did not show any significant confounding of self-perceived recognition ability by demographic or professional GP variables. The GPs who stated that they enjoyed treating patients with MDE, were more likely (p = 0.04) to perceive their recognition ability for MDE as rather good. This constellation was not significant in relation to GAD.

**Table 3:** Demographic and professional characteristics of GPs self-perceived ability to recognize as "moderate/bad" opposed to "rather good"

	MDE			GAD		
	**OR**	**95% CI**	**P**	**OR**	**95% CI**	**P**

Gender: male	1.00					
female	0.77	0.18–3.21	0.71	1.00	0.21–4.77	1.00
Work experience: 12 years	1.00					
> 12 years	0.70	0.20–2.51	0.58	0.29	0.07–1.23	0.94
Number of patients pr week: 100	1.00					
>100	1.14	0.31–4.23	0.84	2.50	0.62–10.11	0.20
Number of seminars last two years : 1						
>1	1.11	0.25–5.04	0.89	1.07	0.21–5.44	0.93
Use of questionnaires or psycho- educational material: seldom	1.00					
often	2.13	0.57–7.99	0.26	0.67	0.16–2.74	0.57
In my opinion MDE and GAD are almost the same: not agree	1.00					
partial or full agree	1.3	0.3–5.2	0.68	1.4	0.3–6.6	0.65
I enjoy treating patients with MDE/GAD not agree	1.00					
partial or full agree	10.0	1.1–93.4	0.04	3.7	0.4 – 33.7	0.25

### Observed ability to recognize severity

**Figure 2:**
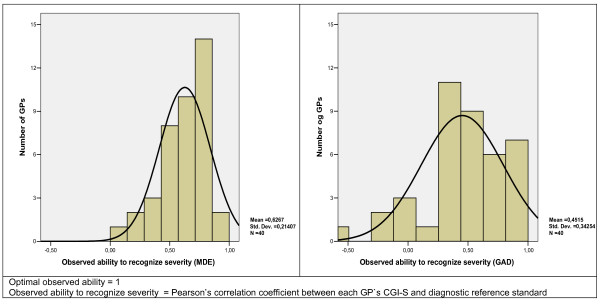
Distribution of observed ability to recognize severity: Major depressive episode and generalised anxiety disorder.

The distribution of the observed ability to recognize severity for MDE and GAD are shown in Figure 2. Pearson's correlation coefficients concerning MDE had a mean of 0.63 (SD 0.21) and for GAD a mean of 0.45 (SD 0.34).

### The association between perceived and observed ability to recognize severity

**Figure 3:**
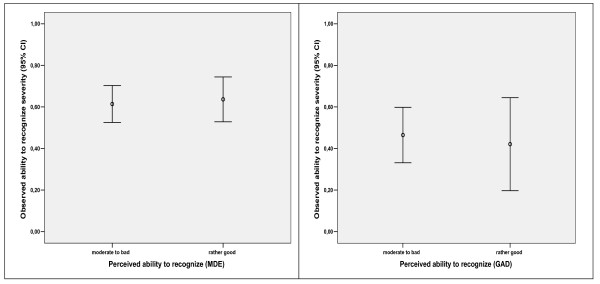
The association between perceived and observed ability to recognize severity.

There were no significant associations between perceived and observed ability to recognize severity, neither in relation to MDE (p = 0.19) nor to GAD (p = 0.34). (Figure 3).

## Discussion

We have three main findings: 1) The sample of GPs characterized their perceived ability to recognize overall as 'rather good' and 'moderate'. 2) The observed ability to recognize severity was moderate (mean correlation coefficients of 0.63 for MDE and 0.45 for GAD). 3) There was no significant association between GPs' perceived and observed ability to recognize severity of MDE or GAD.

The GPs in 55% (22/40) for MDE and in 30% (12/40) for GAD perceived their abilities to recognize these disorders as good. In the German part of the study [21] these frequencies were 64% concerning MDE and 56% for GAD. Concerning MDE the difference in self- perceived ability between Norwegian and German GPs is not significant (p = 0.24). Norwegian GPs do not perceive their own ability as good as the German GPs concerning GAD (p = 0.002), but still only one GP in the Norwegian sample considered his recognition ability 'rather bad' and no one considered it 'bad'. With this high confidence in their recognition abilities, we assume that most GPs' motivation for improvement of their abilities in relation to common mental disorders is only moderate at best.

Efforts to identify variables that characterise GPs with good abilities to recognize MDE and GAD have not given conclusive findings [22]. Neither could we find any demographic characteristics in GPs confounding their self-perceived ability to recognize. However, Dowrick et al [11] reported a significant association between GPs' recognition abilities and their belief in successful treatment.

The problem of under-recognition might be reduced if the GPs think they can identify and treat the symptoms. This could be in accordance with our finding (Table 3) that GPs who enjoy treating depression are more likely to perceive themselves as having rather good abilities to recognize. Another study [23] exposed GPs to 52 patients with validated psychiatric symptoms. The enthusiasm and personality of the GPs determined their positive response to the training program, rather than their years of experience.

The mean coefficient level of the observed recognition ability was 0.63 and 0.45 for MDE and GAD, respectively, which indicate a moderate ability among GPs to recognize a set of diagnostic criteria of common mental disorders. The GPs' moderate ability to recognize these disorders is also indicated by the wide range in the correlation coefficient for MDE (0.92) and for GAD (1.59).

The literature [3,4] on the GPs diagnostic abilities of common mental disorders has mainly focused on under-identification. The agreement between GPs' rates of recognition of mental disorder and diagnostic rating instruments vary considerably depending on the instruments used [24]. Access to information and educational programs to improve knowledge might be useful to reduce the variability in recognition ability. In Norway such educational programs have mainly focused on MDE. The correlation coefficient was higher and showed less variation for MDE than for GAD, and this could imply that these programs have improved recognition abilities among GPs concerning depression. An alternative interpretation could be that MDE is easier to identify than GAD in general practice.

Our study showed a discrepancy between GPs' self-perceived ability to recognize MDE and GAD and their observed ability. We were not able to confirm our hypothesis concerning a significant positive association between GPs' self- perceived recognition ability and their observed ability. This is in accordance with the German part of the study [21] reporting no association between GPs self-perceived and observed ability to recognize. Tracey et al [25] have formerly described low and non-significant correlations between GPs' self-assessed knowledge and test-scores on various non-psychiatric topics. Their study claimed that professional development programmes were likely to fail if they relied on the GPs' self perceived assessments of their educational needs. Explanations of the discrepancy between the GPs' perceived and observed recognition ability could be the rapid inflow of new knowledge within the vast field to be covered by them, or by a busy work load with little opportunity for reading or discussions with colleagues.

Rather than overwhelming GPs with more diagnostic information, helping them to identify lacks in their knowledge could be more useful, for example, by giving them feedback on the discrepancy between self-perceived and observed ability to recognize common mental disorders. A recent Cochrane review [26] assessed the effects on recognition rates by providing GPs with feedback from their patients' scores on screening instruments of depression. Such feedback appeared to be of limited value in improving the GPs' detection and management of depression, however. In the discussion [27] following the paper by Tracey et al. [25], the authors promoted the availability of banks of objective questions that GPs could use for self assessment in order to plan their portfolio of learning each year. Such specified portfolios [28] could contribute to a system ensuring that all doctors took an active part in identifying and meeting their own learning needs. In a multifaceted practice-based prospective design [29] the authors managed to improve recognition rate of depression in general practice, but only if the participating team had the capacity to commit themselves to the program, and a critical mass of the team members was open to change. This finding is in accordance with the review [5] pointing out that educational program ought to be individually tailored. By giving the GPs individually tailored feedback where the discrepancy between their self-perceived and observed recognition ability is one of the issues, the GPs' might get better insight into their current practice and be able to improve their diagnostic performance. However, educators should take into account that the GPs' degrees of insight are variable [30], and that some GPs could be difficult to reach.

### Strengths and weaknesses

The GPs of our study were recruited by a pharmaceutical company, and they agreed to take part in a sponsored study. Several selection biases could be operating. The company marketed an antidepressant (venlafaxine) and therefore, the company could address more GPs who were particularly interested in psychiatry, or who favoured the use of psychopharmacology. Since we do not know if such biases were operating, the representativity of our GP sample and thereby the generalizability of our findings, are somewhat at stake.

A selection bias toward GPs with higher self-confidence concerning their ability to recognize could be operating. In that case we should expect that the bias lead to a narrower range of recognition ability. To these biases we can object that our GPs sample was geographically spread, and had a working experience and gender distribution in accordance with GPs in Norway in general [31]. Patients' age and gender were representative for patients attending GPs in Scandinavia [32], and the prevalence of MDE and GAD reported by them were in accordance with other studies from primary care [3,4].

Although the GPs were representative in relation to gender and experience, we do not know how their attitude toward and knowledge about common mental disorder eventually differed from that of Norwegian GPs in general. The GPs were informed that the study focused on MDE and GAD, but did not get any explicit information about our possibility to explore the discrepancy between their perceived and observed recognition ability.

A limitation of our study was that the scale measuring the GPs' self-perceived ability to recognize common mental disorder had not been psychometrically tested in any of the participating countries before the study started. We can however argue that the face value of the question is better than in the only former study [11] investigating GPs self-confidence in identifying depression. The validity and 'objectivity' of the measure utilised as observed recognition ability might be a subject of discussion. The measure was computed as the mean correlation coefficient between the GPs' CGI-S ratings and the patient-rated DSQ and GAS-Q. CGI-S is well documented as a valid dimensional measure of severity of disorder [20]. The DSQ and the GAS-Q are used as "gold-standards" in a validation study [33] and as the internal consistencies were high (Cronbach's alpha 0.87 and 0.91, respectively), the validity is considered sufficient for the question in concern.

The bonus paid by the pharmaceutical company for each included patient independent of any monitoring of their work, might invite to a sloppy diagnostic practice just for money, leading to increased discrepancy between self-perceived and observed ability to recognize. However, since the influence of the company was restricted to logistics, and the GPs were quite experienced, we assume that our findings do not deviate significantly from the GPs' practice in general.

The patients participating also got the information that the study focused on GAD and MDE. They might have worried for the stigma of getting a diagnosis and as a result could have under-rated their symptoms. Under-estimation of symptom load in this design where the patients' ratings represent the reference standard, and the presumed under recognition by the GPs, could lead to inflation of the observed recognition ability. We found a moderate observed ability, and presumed that the design with regard to informed patients did not have any significant impact on our findings.

We consider it a strength that the GPs were blind to the patients' ratings, when making their severity assessments and that no pre-study training courses inflated the observed recognition ratings. Both the patients' self-ratings and the GPs' ratings of CGI-S are dimensional measures, and this is considered as a methodological strength because both of them imply patients' symptom load and severity of disorders. However, by using the CGI-S it is implicit that GPs are familiar with both mild and severe cases of MDE and GAD, even though that may not be the case. Our findings became statistically more robust by excluding the GPs that examined < 15 patients.

It might be considered a weakness that we based our diagnoses of MDE and GAD on questionnaires rather than on structured psychiatric interviews that could have improved the diagnostic reliability. For logistic and resource reasons interviews could not be applied. It is difficult to know the difference in validity due to the methodological differences between being interviewed for diagnostic criteria rather than self-rating them on a questionnaire. An interviewer could introduce observer bias in the interpretation of symptoms, while the patients could show information bias. Comparison of different ways of identifying patients with mental disorders in general practice has highlighted the complexity of recognition of clinically significant disorders [24].

## Conclusion

This study found a discrepancy between the GPs' perceived and observed ability to recognize common mental disorders. The lacking association between GPs' perceived and observed ability to recognize indicate low understanding of their own recognition abilities. This might contribute to explain the low effect of interventions aimed to increase abilities to recognize mental disorders in GPs.

## Abbreviations

CGI-S: Clinical Global Impression-Severity

CIDI: The Composite International Diagnostic Interview

DSM-IV: American Psychiatric Association. Diagnostic and statistical manual of mental disorders, 4^th ^edition

DSQ: The Depression Screening Questionnaire

GAD: Generalized anxiety disorder

GAS-Q: The Generalized Anxiety Questionnaire

GPs: General practitioners

MDE: Major depressive episode

## Competing interests

The author(s) declare that they have no competing interests.

## Authors' contributions

IO conceived and planned the study, did all the data handling and statistical analyses, and drafted several versions of the manuscript. AM helped designing of the study, supervised the statistic calculations, and drafted the manuscript. AAD was a consultant to Wyeth Ltd Norway in the design of the Norwegian part of the study, participated in the design of the study, and supervised the development of the manuscript. All authors read and approved the final manuscript.
